# Loss-of-function screening to identify miRNAs involved in senescence: tumor suppressor activity of miRNA-335 and its new target CARF

**DOI:** 10.1038/srep30185

**Published:** 2016-07-26

**Authors:** Yue Yu, Ran Gao, Zeenia Kaul, Ling Li, Yoshio Kato, Zhenya Zhang, Joanna Groden, Sunil C Kaul, Renu Wadhwa

**Affiliations:** 1 Drug Discovery and Assets Innovation Lab, DBT-AIST International Laboratory for Advanced Biomedicine (DAILAB), Biomedical Research Institute, National Institute of Advanced Industrial Science & Technology (AIST), Tsukuba-305 8565, Japan; 2 Graduate School of Life & Environmental Sciences, University of Tsukuba, Japan; 3 Institute of Laboratory Animal Science, Chinese Academy of Medical Science (CAMS) & Comparative Medicine Center, Peking Union Medical College (PUMC), China; 4 Department of Molecular Virology, Immunology & Medical Genetics, The Ohio State University, Columbus, Ohio 43210, USA

## Abstract

Significance of microRNAs (miRs), small non-coding molecules, has been implicated in a variety of biological processes. Here, we recruited retroviral insertional mutagenesis to obtain induction of an arbitrary noncoding RNAs, and coupled it with a cell based loss-of-function (5-Aza-2′-deoxycytidine (5Aza-dC)-induced senescence bypass) screening system. Cells that escaped 5-Aza-dC-induced senescence were subjected to miR-microarray analysis with respect to the untreated control. We identified miR-335 as one of the upregulated miRs. In order to characterize the functional significance, we overexpressed miR-335 in human cancer cells and found that it caused growth suppression. We demonstrate that the latter accounted for inhibition of 5-Aza-dC incorporation into the cell genome, enabling them to escape from induction of senescence. We also report that CARF (Collaborator of ARF) is a new target of miR-335 that regulates its growth suppressor function by complex crosstalk with other proteins including p16^INK4A^, pRB, HDM2 and p21^WAF1^.

MicroRNAs (miRs) are a class of highly conserved small non-coding molecules (about 21–25 nucleotides long) that act as gene repressors by either causing their mRNA degradation or translational block. They are transcribed as pri-miRNAs and are subsequently processed into short hairpin structured molecules by Drosha, the double-stranded RNA specific ribonuclease. Their involvement in diverse biological processes ranging from normal development to a variety of pathogenesis has been implicated. Hence, miR profiling has been considered to yield valuable outcomes, not only to understand the regulation of basic biological phenomena but also, in disease, diagnosis, therapy and prognosis^1^,^2^,^3^.

Cancer is a complex disease. It is regulated by multifaceted network of signaling pathways driven by loss of activities of tumor suppressor proteins, gain of function of oncogenes and several epigenetic mechanisms^4^,^5^,^6^. Many miRs, including miR-21, miR-143, miR-145, miR-182, have been found as enriched in tumors and shown to possess oncogenic functions^7^,^8^,^9^. On the other hand, several others including, miRNA-125b, miR-335-5p, and miR-34 family are downregulated in several types of cancers^10^,^11^,^12^,^13^,^14^. Recently, it was shown that the tumor suppressor protein p53 regulates miRNA expression and processing, and in turn gets regulated by miRs. Restoration of p53-induced miRs was shown to cause suppression of tumor growth and metastasis in mouse models of cancer suggesting that there is a complex network of miR-p53 interactions in the regulation of p53 activities, its effectors and regulators^15^,^16^,^17^. Several other tumor suppressor pathways including pRB, PTEN, p16^INK4A^, BMI and p14^ARF^ have been shown to be either regulated by miRs or involve them for their activities to implement control on cell proliferation^18^,^19^,^20^,^21^,^22^. In spite of imperative emerging evidence of the role of several miRs in cancer, the molecular targets and mechanisms remain largely undefined. In addition, a number of miRs have also been characterized to possess both tumor suppressor and oncogenic functions^23^ warranting molecular insights to their activities in context of cell physiology.

Epigenetic control of cancer has been well-established^6^. Several epigenetic drugs (DNA methyltransferases and histone deacetylase inhibitors) that induce senescence in cancer cells have been in practice in conventional chemotherapy. However, their impact on the miRs and cancer progression remains largely unknown^24^. It was reported that miR-34 and miR-145 were silenced through DNA hypermethylation and were, hence, induced by 5-Aza-dC treatment^25^,^26^. In the present study, we used a retroviral vector containing GFP reporter to introduce retroviral insertional mutagenesis^27^. Genomic integration of this vector was expected to induce transcription of sequences downstream to its integration sites. Based on the fact that more than 98% of the human genomic DNA constitutes protein-noncoding sequences, such random integration of the vector in genome was expected to induce a large variety of miRNAs (referred to as arbitrary miR library). Cells expressing such random library of miRs were subjected to 5-Aza-dC induced senescence for 3–5 days. Whereas untransduced controls showed senescence phenotype, some virus-transduced cells escaped from 5-Aza-dC induced senescence (called loss-of-function/senescence bypass). These cells were subjected to miR-array data analysis with respect to the control (untransduced) cells. Out of several upregulated miRs, we characterized the function of miR-335 in the present study. We report that miR-335 possesses tumor suppression function, mediated, at least in part, by targeting CARF that in turn regulates several cell cycle monitoring proteins including p16^INK4^, pRb, p53 and p21^WAF1^.

## Results and Discussion

### Identification of miR-335 in 5Aza-dC induced senescence bypass

**Figure 1:**
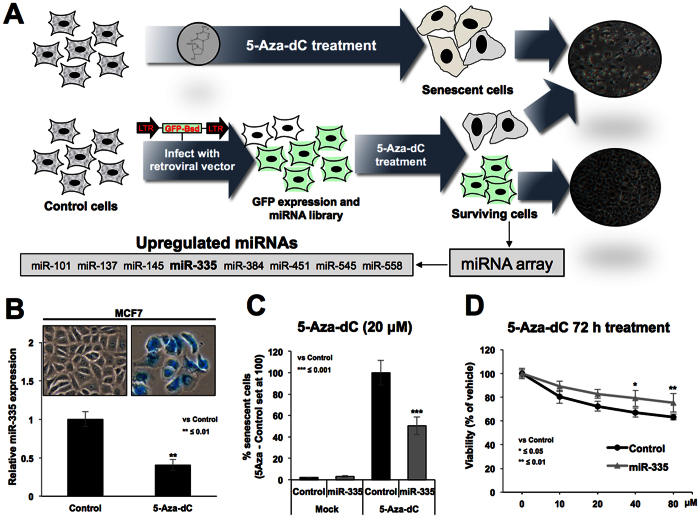
Identification of miR-335. Strategy for induction of miR library in human cancer cells and loss-of-function/senescence bypass screening is described (**A**). The cells infected with bicistronic vector constituting of two promoters and GFP were treated with 5-Aza-dC. Whereas control uninfected cells showed induction of senescence, colonies emerged in the virus harboring cells. These colonies were expanded and pool of colonies was subjected to miR-array analysis with respect to the untransduced control cells. miR array analysis resulted in identification of miR-335 as one of the upregulated miR in cells that escaped 5-Aza-dC-induced senescence. Cells undergoing 5-Aza-dC-induced senescence showed down-regulation of miR-335 (**B**). Cells overexpressing miR-335 showed resistance to 5-Aza-dC-induced senescence as examined by senescence associated β-gal staining (**C**) as well as cell viability assays (**D**).

A retroviral vector constituting two long terminal repeat (LTR) promoters at 5′ and 3′ ends of GFP gene was generated in a way that the random integration of this vector in the genome would result into (i) expression of GFP; detected by green fluorescence and (ii) its integration site-dependent arbitrary manipulation of the host cell genome. The latter may yield loss-of-function phenotype due to altered expression of either proteins or the noncoding microRNA regulators. We coupled this system with induction of senescence in human cancer (U2OS) cells by demethylating drug, 5-Aza-dC (Fig. 1A). Cells treated with 5-Aza-dC showed strong induction of cellular senescence. More than 90% of control (virus untransduced) cells showed growth arrest and senescence associated β-gal staining in about 3–5 days. Small colonies appeared in virus-transduced cells at about 4–6 days. These showed green fluorescence, and hence were originated from transduced cells that bypassed senescence by loss of response to 5Aza-dC. The cells were expanded and were subjected to microRNA array with respect to the untransduced and untreated control cells (Fig. 1). We found several miRNAs (miR-101, miR-137, miR-145, miR-335, miR-384, miR-451, miR-545 and miR-558) upregulated in virus-transduced cells that showed resistance to 5-Aza-dC-induced senescence (Fig. 1 and Supplementary Fig. 1A,B). Of note, two of these miRs (miR-145 and miR-335) have been reported to undergo hypermethylation-mediated silencing in a large variety of cancers^25^,^28^. Hence, their upregulation by 5-Aza-dC induced demethylation was justified. Furthermore, it may also be the result of retroviral insertional mutagenesis^27^ (induction of transcription downstream of integration of the virus to cell genome). These data suggested that these miRs are involved in regulation of induction of cellular senescence, proliferation and drug response of cells. In the present study, we aimed to characterize such functions and targets of miR-335.

### miR-335 compromises 5-Aza-dC induced senescence by inducing growth suppression

In view of the literature that miR-335 is silenced by hypermethylation in cancer cells^25^,^28^, we first examined if induction of senescence by 5-Aza-dC involved upregulation of miR-335 expression. Untransduced control cells were treated with 5-Aza-dC. Induction of senescence was tracked by examining the cell morphology and number in control and treated cells. At about 5–6 days post-treatment, treated cell cultures showed senescent morphology and growth arrest, also confirmed by senescent associated β-gal staining (Fig. 1B). Senescent cells were collected and subjected to miR-335 expression analysis. Contrary to our expectation, we found that miR-335 was downregulated in treated cells (Fig. 1B) suggesting that its upregulation, as detected by miR-array analysis, may have role in escape from senescence. An expression plasmid encoding primary miR-335 was transfected into U2OS cells. Treatment of control or miR-335-transfected cells with 5-Aza-dC and detection of senescent cells by senescence associated β-gal staining revealed that prior upregulation of miR-335 indeed compromised induction of senescence. There was about 50% reduction in the number of SA-β-gal positive cells in miR-335 transfected cells (Fig. 1C). Cell viability analysis also showed that miR-335 transfected cells were less responsive to 5-Aza-dC, particularly at doses higher than 20 μM (Fig. 1D) suggesting that prior upregulation of miR-335 was involved in the escape from 5-Aza-dC induced senescence/growth arrest. Taken together, these data suggested that miR-335 was downregulated in cells that showed senescence upon 5-Aza-dC treatment, and that the overexpression of miR-335 suppressed such effect of 5-Aza-dC.

**Figure 2:**
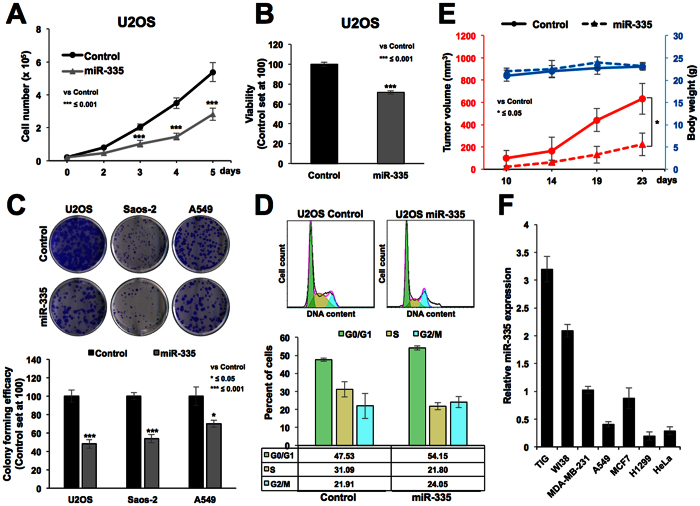
miR-335 overexpression caused growth suppression of cancer cells *in vitro* and *in vivo*. Growth curve of control and miR-335 overexpressing cells showed slower growth of the latter (**A**). Short and long term survival by viability analysis (**B**) and colony forming assay (**C**) showed about 30% and 50% reduction, respectively. Cell cycle analysis revealed increase in number of cells at G0/G1, G2/M and decrease in S phase (**D**). miR-335 overexpressing cells, in subcutaneous xenografts in nude mice, showed low tumor forming capacity as compared to the control cells. Body weight of the mice during the course of experiment showed no difference (**E**). Expression analysis of miR-335 in human normal and cancer cells showed its higher level of expression in normal cells (**F**).

**Figure 3:**
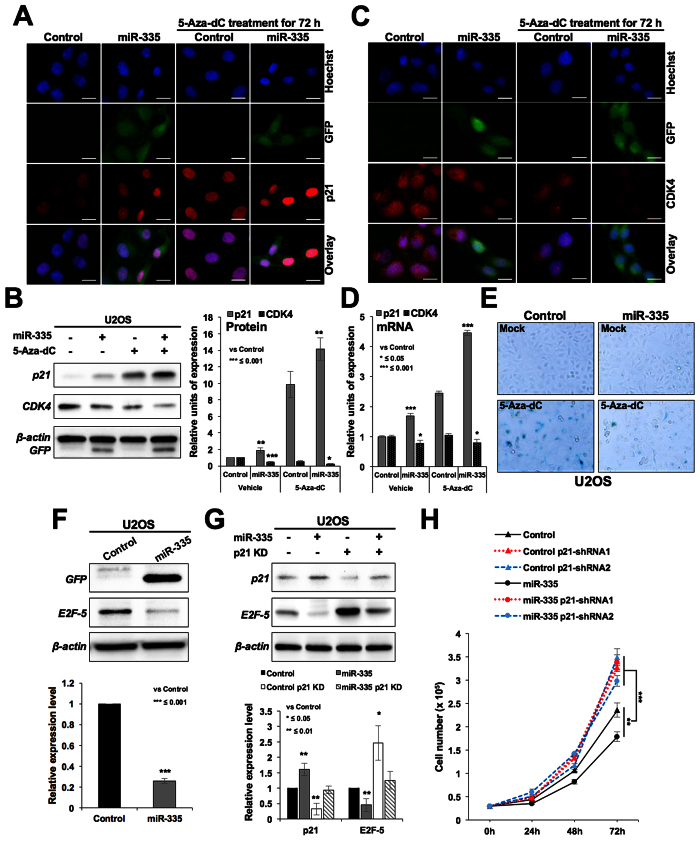
miR-335 overexpression mediated growth suppression involves upregulation of p21^WAF1^. miR-335 overexpressing cells showing growth suppression possessed higher level of expression of p21^WAF1^ (**A,B**) and low level of expression of CDK4 (**C,B**). mRNA expression revealed increased level of p21^WAF1^ and decreased level of CDK4 in miR-335 overexpressing cells (**D**). miR-335 overexpression caused growth suppression *per se*, and resistance to 5-Aza-dC induced senescence (**E**). miR-335 overexpressing cells showed downregulation of E2F5 (**F**) that was abolished by simultaneous knockdown of p21^WAF1^ using p21^WAF1^ specific shRNA-1 (**G**). Retardation of cell growth in miR-335 transfected cells was abrogated by shRNA mediated knockdown of p21^WAF1^ (**H**). Scale bars represent 20 μm.

In order to clarify the mechanism of the role of miR-335 in escape from 5-Aza-dC induced senescence, we considered two possibilities: (i) based on the fact that incorporation of 5-Aza-dC takes place during replication and DNA synthesis, we hypothesized that miR-335 might cause growth arrest of cells and thus do not allow 5-Aza-dC to be incorporated into the genome, and (ii) miR-335 may target genes that are essential for 5-Aza-dC induced growth arrest. We tested the first possibility by analyzing the growth and cell cycle characteristics in control and miR-335 transfected cells. As shown in Fig. 2, the latter were retarded in their growth (A) and showed lower viability in short term (B) as well as long term colony-formation assays (C). Cell cycle analysis revealed increase in number of cells at G0/G1, G2/M and decrease in S phase (Fig. 2D). These data suggested that miR-335 suppressed the growth of cells that may have contributed, at least in part, to escape of cells from 5-Aza-dC induced senescence, as hypothesized above. miR-335 induced suppression of cell growth was also confirmed by *in vivo* subcutaneous xenografts of control and miR-335 transfected A549 cells in nude mice (Fig. 2E). We found that the tumor growth of miR-335 overexpressing cells was suppressed as compared to the control cells. In order to investigate the relevance of miR-335 in proliferation rate of cells, we performed its expression analysis in normal and cancer cells. Amongst different cancer cell lines, we found ~5 fold variation in the level of miRNA. Nevertheless, consistent with its above described growth suppression function, miR-335 expression was ~2–4 fold higher in normal fibroblasts as compared to a variety of cancer cells (Fig. 2F). Furthermore, in line with its growth suppression activity in *in vitro* and *in vivo* assays, the miR-335 overexpressing cells showed upregulation of p21^WAF1^ (Fig. 3A,B and Supplementary Fig. 2A) and downregulation of CDK4 at protein (Fig. 3C,B and Supplementary Fig. 2B) and mRNA (Fig. 3D) levels. Furthermore, as expected, the growth suppression induced by miR-335 *per se* (Fig. 2A,E) was associated with substantial resistance to induction of growth arrest/senescence by 5-Aza-dC (Fig. 3E). As expected, increase in p21^WAF1^ was associated with decrease in E2F5 in miR-335 overexpressing cells (Fig. 3F). Simultaneous knockdown of p21^WAF1^ by shRNA abolished miR-335-induced decrease in E2F5 (Fig. 3G) resulting in recovery, at least in part, from growth suppression caused by miR-335 (Fig. 3H). These data suggested that p21^WAF1^ plays a key effector role in miR-335-induced growth suppression signaling.

### Effect of miR-335 on p16^INK4A^ and pRB, resulting in compromised 5-Aza-dC-induced senescence

**Figure 4:**
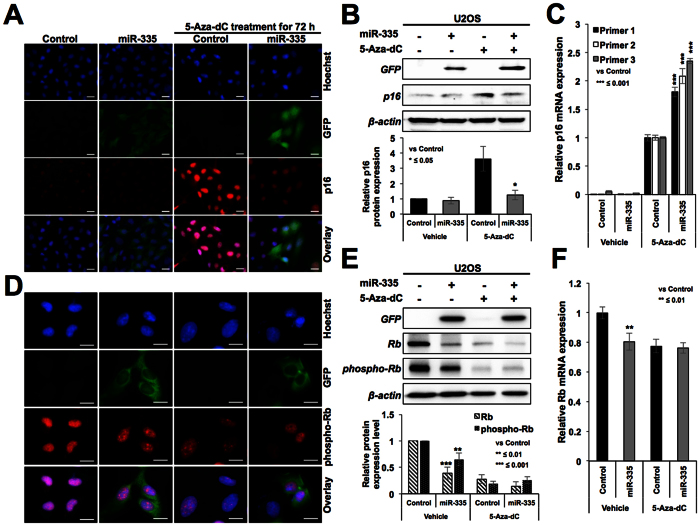
miR-335 overexpressing cells showed downregulation of p16^INK4A^ and pRB. Immunostaining (**A**) and Western blotting (**B**) of control, miR-335-transfected cells treated with 5Aza-dC showed that the latter caused increase in expression of p16^INK4A^ and miR-335 compromised p16^INK4A^ increase at the protein (**A**,**B**) but not mRNA level (**C**). miR-335 transfected and 5Aza-dC treated cells showed increase in p16^INK4A^ transcript that was confirmed by three sets of primers. Immunostaining (**D**) and Western blotting (**E**) for pRB in control and miR-335 cells revealed low level of expression of pRB and pRB^phospho^ in the latter. miR-335 transfected cells showed lower level of pRB transcript (**F**). Scale bars represent 50 μm in A and 20 μm in B.

We next considered the second possibility that the miR-335 may target essential genes involved in 5-Aza-dC induced growth arrest. In U2OS cells, p16^INK4A^ (cyclin-dependent kinase inhibitor and tumor suppressor) is known to undergo silencing by hyper-methylation of its promoter^29^. As expected, 5-Aza-dC treated cells showed induction of p16^INK4A^ (Fig. 4A,B and Supplementary Fig. 2C). Of note, we found that miR-335-transfected cells showed downregulation of 5-Aza-dC-induced p16^INK4A^ protein expression (Fig. 4A) suggesting that miR-335 may target p16^INK4A^ directly/indirectly and hence may account for escape of 5-Aza-dC-induced senescence. Further to confirm, we determined the expression of p16^INK4A^ in control and miRNA-transfected cells by qPCR using three sets of primers and, surprisingly, found increase in p16^INK4A^ transcript not only in 5Aza-dC-treated controls, but also miR-335 overexpressing cells (Fig. 4C). These data suggested that the decrease in p16^INK4A^ protein observed in miR-335 overexpressing cells (Fig. 4A,B) was not due to direct targeting of p16^INK4A^ mRNA by miR-335.

p16^INK4A^ is an established inhibitor of cyclin D-CDK4 complex. The latter phosphorylates retinoblastoma protein resulting in its dissociation from E2F family of proteins that are required for cell cycle progression. Accordingly, decrease in p16^INK4A^ in miR-335 overexpressing cells was expected to result in activated cyclin D-CDK4 complex resulting in increased level of phosphorylated pRB in these cells. We examined the levels of pRB and phospho-pRB and found, contrary to the expected, decrease in phospho-pRB in miR-335 derivatives (As shown in Fig. 4D and Supplementary Fig. 2D). Western blotting with pRb and phospho-pRB specific antibodies also confirmed their downregulation in miR-335 transfected cells (Fig. 4E). qPCR analysis also revealed decrease in pRB transcript in miR-335 overexpressing cells (Fig. 4F) endorsing that miR-335 targets pRB. Such decrease in phospho-pRB was attributed to cause cell cycle retardation and escape of cells from 5-Aza-dC induced senescence. Another study also showed that during differentiation of mouse embryonic stem cells, miR-335 is upregulated and causes downregulation of Oct4-pRB axis and pRB dephosphorylation^19^. In this report, miR-335 was shown to target pRB directly and specifically targeting a conserved sequence motif in its 3′ un-translated region^18^. However, it was shown to cause consequential activation of p53 tumor suppressor resulting in inhibition of cell proliferation and transformation. Taken together with our findings, it emerged that miR-335 is an important miR that controls cell proliferation by balancing the activities of pRB and p53 tumor suppressor pathways.

### miR-335 targets CARF and compromises 5-Aza-dC induced senescence

**Figure 5:**
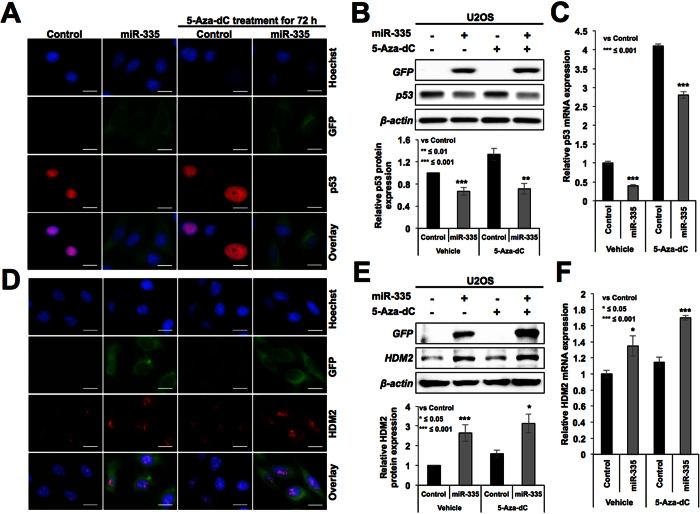
miR-335 overexpressing cells showed compromised expression of p53. Immunostaining (**A**) Western blotting (**B**) and qPCR (**C**) analyses for p53 showed decreased level of expression in miR-335 overexpressing cells. The transfected cells showed higher level of expression of HDM2; immunostaining (**D**) Western blotting **(E**) and qPCR (**F**) data are shown. Scale bars represent 20 μm.

In light of the information that miR-335 targets pRB and activates p53^18^, we examined the level of p53 in control and miR-335 transfected cells. As shown in Fig. 5, we found decrease in protein as well as mRNA level of p53 in miR-335 transfected cells (Fig. 5A–C and Supplementary Fig. 2E). The data suggested that miR-335 targets p53 and contributes to escape of cells from 5-Aza-dC-induced senescence. However, as shown in Fig. 3B, downstream effector of p53, p21^WAF1^, showed increase in miR-335-overexpressing cells. We examined the HDM2 protein, another downstream effector and antagonist of p53, by immunostaining and Western blotting, and found its elevated level in miR-335-transfected cells (Fig. 5D,E and Supplementary Fig. 2F). These results were also supported by qPCR analysis (Fig. 5F) suggesting that miR-335 may target an upstream inhibitor of HDM2, leading to its increased levels that in turn may contribute to decreased level of p53 in miR-335 transfected cells.

**Figure 6:**
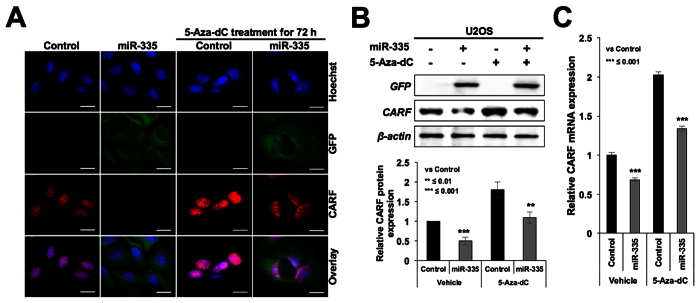
miR-335 overexpressing cells showed decrease in CARF expression. Immunostaining (**A**) Western blotting (**B**) and qPCR (**C**) analysis for CARF showed decreased level of expression in miR-335 overexpressing cells. Scale bars represent 20 μm.

CARF (Collaborator of ARF), a protein that poses two-way control on cell proliferation, has been shown to be an upstream regulator of p53^30^,^31^,^32^. Whereas its upregulation occurred during replicative and premature senescence, its super-high level was shown to be associated with tumorigenesis and malignant transformation of cancer cells^33^. It was shown to act as a transcriptional repressor of HDM2^34^. In view of our above findings, we examined CARF-p53-HDM2-p21 axis in miR-335 overexpressing cells. As shown in Fig. 6A–C and Supplementary Fig. 2G, these cells showed down-regulation of CARF at both protein as well as mRNA levels implying that miR-335 targets CARF, and may account for increased level of HDM2 and decreased level of p53 (Fig. 5A,D, respectively). Vector transfected cells did not show decrease either in CARF or in p53 (Supplementary Fig. 1C).

**Figure 7:**
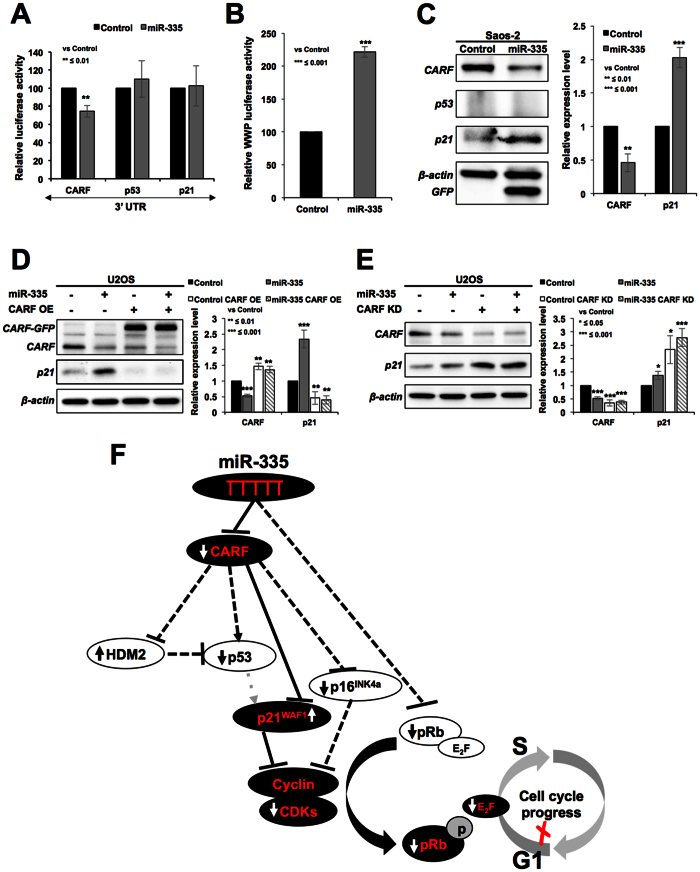
miR-335 targets CARF, not p53. 3′UTR reporter assay for CARF, p53 and p21 in control and miR-335 overexpressing cells showed that miR-335 targets CARF (**A**). p21^WAF1^ promoter reporter assay in control and miR-335 transfected cells showed upregulation of p21 (**B**). Increase in p21^WAF1^ occurred in p53−/− cells and was hence independent to that of p53 (**C**). Overexpression of CARF compromised miR-335 induced downregulation of p21^WAF1^ (**D**). siRNA-mediated knockdown of CARF strengthened the miR-335 induced increase in p21^WAF1^ (**E**). Schematic presentation of miR-335 targets as resolved in this study. miR-335 targets CARF and pRB (**F**). Dominant pathways are shown by solid lines. Upregulation and downregulations of proteins are shown by upward and downward arrows. Decrease in CARF caused (i) increase in HDM2 resulting in decrease in p53 and (ii) increase in p21^WAF1^ causing decrease in CDK4 and E2F5 (as shown in Fig. 3B,F, respectively) resulting in delay in cell cycle progression (shown in Figs 1 and 2).

In order to finally resolve whether miR-335 directly targets CARF, p53 and p21^WAF1^, we performed reporter assays using 3′UTR regions of these genes. As shown in Fig. 7A, we found that CARF, but not p53 and p21^WAF1^, was the target of miR-335. Of note, target site search for miR-335 on CARF, p53 and p21^WAF1^ 3′UTR gene sequences predicted two sites in CARF (Supplementary Fig. 1D), and none in the p53 and p21^WAF1^ 3′UTR. These data supported our above findings, and excluded p53 and p21^WAF1^ to be a target of miR-335. This was consistent with the findings of Scarola *et al*.^18^ who reported that p53 was not the target of miR-335. Instead, it showed increase that was consequent to decrease in pRB in cells transiently transfected with miR-335. We also found increase in p53 in cells transiently transfected with miR-335 (Supplementary Fig. 1E). However, stable transfections of miR-335 caused decrease in p53 (Fig. 5A–C and Supplementary Fig. 1E) that was attributed to increased level of HDM2 (Fig. 4D–F) (an established antagonist of p53) that is normally repressed by CARF^34^. Of note, CARF has been shown to act as transcriptional repressor of p21^WAF1 ^35 and hence targeting of CARF by miR-335 also accounted for upregulation of p21^WAF1^ (Fig. 3A,B) that occurred contrary to the decreased level of expression of p53. p21^WAF1^ promoter luciferase reporter assays in control and miR-335-transfected cells also showed increase in p21^WAF1^ reporter activity in the latter (Fig. 7B). Furthermore, we determined that this increase in p21^WAF1^ was independent to that of p53 by using p53−/− cells (Saos-2). As shown in Fig. 7C, miR-335-transfected cells showed decrease in CARF and increase in p21^WAF1^suggesting that miR-335 targets CARF and caused increase in p21^WAF1^ independent to that of p53. In order to further validate, we reconstituted CARF in miR-335 transfected cells and found that an overexpression of CARF abolished the increase in p21^WAF1^ transfected cells (Fig. 7D). siRNA-mediated CARF knockdown, on the other hand, potentiated the effect of miR-335 on p21^WAF1^ expression (Fig. 7E).

CARF is an essential cell proliferation-regulatory protein that is upregulated during replicative and stress-induced senescence. Suppression of CARF induced aneuploidy, DNA damage and mitotic catastrophe resulting in apoptosis endorsing that it is an essential for cell survival^31^. It has been shown to bind to multiple proteins, p14^ARF^, p53 and HDM2 of ARF-p53-p21 tumor suppressor axis, and control cancer cell proliferation in two directions in a dose dependent way^30^,^33^,^36^. It was shown that while moderate level of CARF overexpression induced senescence, its very high level resulted in increased cell proliferation. Hence, CARF was demonstrated to determine the proliferative fate of cancer cells towards growth arrest, or pro-proliferative and malignant phenotypes by feedback and feed-forward regulatory loops of CARF-ARF-p53-HDM2-p21^WAF1^ axis. Molecular mechanism(s) of such dual regulation and how CARF is regulated in normal and cancer cells have not been fully understood. It was shown that CARF is involved in drug-induced senescence^30^. In the present study, we found that the cells induced to senescence (confirmed by SA-β-gal staining) in response to treatment with 5-Aza-dC for 5–7 days showed decrease in miR-335 (Fig. 1B). Such decrease may account for moderate increase in CARF and senescence through activation of p53^30^,^31^,^33^,^36^. On the other hand, prior increase in miR-335 (due to retroviral insertional mutagenesis) and targeting of CARF activated p21^WAF−1^ and resulting in growth arrest (Fig. 7B). Such prior induction of miR-335-induced growth arrest would inhibit the incorporation of 5-Aza-dC into the genome and account, at least in part, for escape of cells from 5-Aza-dC-induced senescence.

In agreement with our findings on identification of miR-335 in retrovirus-induced arbitrary manipulation of genome and cell based functional screening of miRs involved in escape from 5-Aza-dC induced senescence, Gao *et al*.^13^ and Dohi *et al*.^28^ have also reported that miR-335 expression was increased after 5-Aza-dC treatment, endorsing its regulation by methylation. Deletion and hypermethylation of microRNA-335 locus (7q32.2) has been reported in patient-derived metastatic breast and ovarian cancer cells. Its restoration inhibited migration via targeting extracellular matrix glycoprotein (Tenascin-C) involved in cell migration^37^, and was shown to induce premature senescence in young mesangial cells via downregulation of SOD2 and Txnrd2 with a concomitant increase in reactive oxygen species (ROS)^38^. It was also suggested as a strong inhibitor of tumor re-initiation^39^. Downregulation of miR-335 in a variety of tumors and its tumor suppressor function has recently been emerged in several independent studies that have also demonstrated its multiple gene targets^40^,^41^. These include RANKL and IGF-IR in SCLC^42^, BRCA1-regulatory cascade and c-Met protein in breast cancers^13^, OCT4 in pancreatic cancer^43^, PAX6 in glioma^44^, Bcl-w or BCL2L2 in the ovarian cancer^45^, RUNX2 in hMSCs^40^, ROCK1 and MAPK1 in neuroblastoma^46^, Bcl-w and specificity protein 1 (SP1) in gastric cancer^14^,^47^ and ZEB2 in colorectal cancer^48^.

We demonstrated that CARF is a new target of miR-335 that mediates its tumor suppressor function (Fig. 7F). Of note, CARF silencing by shRNA has earlier been shown to cause tumor suppression in nude mice tumor formation assays^36^, in line with tumor suppression obtained with miR-335 in the present study (Fig. 2E). Pleotropic effects of miR-335 demonstrated in the present study (Fig. 7F) and others (as discussed above) may be due to, at least in part, its control on CARF expression that has been shown to impose a dose dependent and contrasting effect on proliferation and malignant transformation of cancer cells^33^. In the present study, we demonstrated that targeting of CARF by miR-335 resulted in upregulation of p21^WAF1^ and downregulation of cyclin-CDK activity (Fig. 3). Furthermore, CARF has also been shown to cause upregulation of p16^INK4A ^31. Hence CARF-targeting by miR-335 also accounted for downregulation of p16^INK4A^ protein (Fig. 4A,B) in spite of the fact that p16^INK4A^ mRNA was increased in miR-335 and 5-Aza-dC treated cells (Fig. 4C). These data suggested miR-335 effects (such as, decrease in p16^INK4A^ protein, increase in HDM2 and p21^WAF1^) and outcomes such as growth arrest are determined predominantly by CARF-targeting. Taken together, CARF emerged as a new strong target of miR-335 and warrant further studies for its potential as an important noncoding tumor suppressor in cancer therapeutics.

## Materials and Methods

### Cell culture and drug treatment

Human osteosarcoma (U2OS and Saos-2), cervical carcinoma (HeLa), breast adenocarcinoma (MCF7 and MDA-MB-231), lung carcinoma (A549 and H1299) and normal human fibroblasts (TIG, MRC5 and WI38) were purchased from Japanese Collection of Research Bioresources (JCRB, Japan) and cultured in Dulbecco’s modified Eagle’s medium (DMEM; Gibco BRL, Grand Island, NY, USA) supplemented with 10% (v/v) fetal bovine serum (Gibco BRL), and 1% (v/v) penicillin/streptomycin in the presence of 5% CO_2_ at 37 °C as described earlier^30^,^31^,^32^,^33^,^34^,^35^. Cells were treated with 20 μM of 5-Aza-2′deoxycytidine (5-Aza-dC) (Sigma-Aldrich, St. Louis, MO, USA) for 72–96 h and subjected to the following *in vitro* assays.

### Retrovirus infection

Exogenous expression of CARF was carried out using a retroviral carrier of GFP-tagged CARF that was cloned into a pCX4neo vector as previously described^33^ All the transfections were performed with FuGENE6 (Roche Applied Sciences, Basel, Switzerland), following the manufacturer’s protocol. Culture medium was replaced with fresh media 24 h after transfection. The viral stock was diluted 1/100, supplemented with 8 μg/ml polybrene and used to infect cells for the generation of CARF overexpressing cells. Selection of infected clones was performed with medium containing G418 (500–900 μg/ml) after 18–24 h to obtain stable GFP-CARF expressing cell lines.

### RNA interference (RNAi) and plasmid transfections

The synthesis and sequences of CARF siRNAs are described elsewhere^30^. Briefly, for annealing of siRNAs, 20 μM of each control or target sense and antisense strand were incubated in annealing buffer (100 mM potassium acetate, 30 mM HEPES-KOH at pH 7.4 and 2 mM magnesium acetate) for 1 min at 90 °C followed by 1 h at 37 °C. siRNA duplexes was carried out using lipofectamine RNAiMAX (Invitrogen, Carlsbad, CA, USA), following the manufacturer’s protocol. Two p21 shRNA vectors (target sites, shRNA-1: agagttgattgaattcaac and shRNA-2: atagatttctatcatttca) were used as described earlier^49^. The expression or shRNA vectors were transfected into cells using X-tremeGENE 9 DNA transfection reagent (Roche), following the manufacturer’s protocol.

### Induction of arbitrary miR library and screening for miRNAs involved in escape from 5Aza-dC induced senescence

To construct a retroviral vector pMXGbu, a short linker with 5′- gaattAGCGGAGGACAGTACTCCGATCGGAGGACAGTACTCCGTtcgac -3′ was inserted between *EcoR*I and *Sal*I sites of pMXCRGb^50^ by removing CMV-RFP cassette. Upon transduction, the upstream LTR of the vector drives GFP-Bsd fusion gene and the downstream LTR initiates random transcription resulting into retroviral insertional mutagenesis^27^ and thus arbitrary alteration of gene expression. The transduced cells were selected in blasticidin (10 μg/ml) supplemented medium and were then subjected to 5Aza-dC (20 μM) for 3–5 days. The cells were subsequently harvested to prepare total RNA (RNeasy Plus Mini Kit (QIAGEN).

### miRNA array

Control (untransduced and untreated) and virus transduced and 5-Aza-dC (20 μM) treated cells (pooled colonies) that escaped senescence were harvested. For miRNA microarray, small RNAs (less than 200 nt including precursor and mature miRNAs) were extracted using mirVana miRNA isolation kit (Ambion, Austin, TX, USA) following the manufacturer’s protocol. Purified RNA was labeled with Cy3 or Cy5 using the mirVana miRNA labeling kit (Ambion). Labeled RNA was hybridized with oligonucleotides against human miRs (~500) arrayed on slides (Hokkaido-System Science, Japan), and detected by a scanner (Agilent Technologies, Santa Clara, CA, USA). miRs that showed more than 2 fold change in expression were considered for further analysis.

### Cloning of miR-335, expression plasmid

pCXGb-miR-335: a primary miR-335 region was amplified from human genomic DNA by PCR using the following primers: 5′-AACTCGAGTTCAGCCTTCATTGTTTAATCTTTACAACAGC-3′ and 5′- AAGATATCTGTATGGACATGAAGCTTTTACTTCAACATTAG-3′. The PCR product was digested with *Sal*I and *Eco*RV and introduced into pCXGb as described earlier^51^.

### Senescence-associated β-galactosidase assay (SA-β-gal)

SA-β-gal detection kit (Cell Signaling Technology, Danvers, MA, USA) was used following manufacturer’s instructions. Cells showing blue staining were considered positive and counted under the microscope quantitation from three independent experiments was performed.

### Flow cytometry

Control and transfected cells were harvested by trypsin (0.25%). Cell pellets were washed with cold PBS and then added drop-wise to pre-chilled 70% ethanol. The fixed cells were centrifuged at 800 X*g* for 5 min at 4 °C, washed with cold PBS twice and then re-suspended in 0.25 ml PBS. The cells suspensions were treated with RNase A at 37 °C for 1 h followed by brief centrifugation to discard supernatant. Cells were re-suspended in 200 μl of Cell Cycle Guava reagent (Millipore, Billerica, MA, USA), incubated for 30 min in dark, and analyzed by EasyCyte Guava cytometer (Millipore). The data were further analyzed using FlowJo software.

### Cell viability, proliferation and colony forming assay

Equal number of control and transfected cells were plated in 96-well plates. After 48 h of incubation at 37 °C, 100 μl of MTT (Sigma-Aldrich) in PBS (5 mg/ml) was added to each well. After 4 h of incubation at 37 °C, the supernatant was discarded and the precipitate was dissolved with 100 ml of dimethyl sulfoxide (DMSO). Plates were then read on a microplate reader (infinite M200 PRO, TECAN) at 570 nm.

For cell proliferation assay, control and transfected cells were plated onto 12-well plates. Cells were harvested and counted in triplicates at the indicated time point using TC20^TM^Automated Cell Counter (Bio-Rad, Hercules, CA, USA), with trypan blue exclusion to identify viable cells. Growth curves were generated for each cell line from three independent experiments.

For long-term cell viability and proliferation, colony-forming assay was performed. 500 cells were plated in a 6-well plate and left to form colonies for the next 10–15 days with a regular change of medium on every third day. Colonies were fixed with pre-chilled methanol/acetone (1/1, v/v) 10 min on ice, stained with 0.1% crystal violet solution, photographed and counted.

### Cloning of pMIR-CARF-3′UTR plasmid

CARF-3′untranslated region (UTR) was amplified by PCR (initial 10 min denaturation step at 98 °C followed by 30 cycles of 98 °C for 30 s, 55 °C for 30 s and 72 °C for 30 s, with a final annealing step at 72 °C for 10 min) using human genomic DNA with primers: 5′-GTTTAAACGTTTAAACTGTGTCCAAAATATCACTGC-3′ and 5′-ACTAGTACTAGTCTAACAGACACGTTCAAC-3′. The PCR product was digested with *Pme*1 and *Spe*1, and then introduced into pMIR-REPORT^TM^ Luciferase plasmid (Applied Biosystems, Forster, CA, USA) between these two sites.

### Luciferase reporter assay

The pGL4-p53-3′UTR and pGL4-p21-3′UTR were generously provided by Dr. Chae-Ok Yun (Hanyang University, Seoul, South Korea). U2OS control and miR-335 transfected cells were transfected with 1 μg of luciferase constructs (pGL4-p53-3′UTR, pGL4-p21-3′UTR or pMIR-CARF-3′UTR), 100 ng of control vector oligonucleotide (pRL-TK or pMIR-REPORT^TM^ β-gal control plasmid) using Lipofectamine 2000 (Invitrogen, Carlsbad, CA, USA). After 24–48 h, luciferase activity was measured using a dual luciferase reporter assay system and β-gal enzyme system (Promega, Madison, WI, USA) following the manufacturer’s protocol. Experiments were carried out in triplicate and repeated at least three times.

### RNA extraction and real-time qRT-PCR

Total RNA was isolated from cells using the RNeasy mini kit (Qiagen, Standford Valencia, CA, USA). The concentration and purity of RNA were determined by ultraviolet spectrophotometry (A260/A280 >1.9) using NanoDrop ND-1000 (Nanodrop Technologies, Wilmington, DE, USA).

Equal amount of RNA were used for reverse transcription following the protocol of QuantiTect Reverse Transcription Kit (Qiagen). The real-time qRT-PCR (50 °C for 2 min; 95 °C for 10 min, 40 cycles of 95 °C for 15 s, 60 °C for 1 min; and 72 °C for 30 s) was performed using SYBR^®^ Select Master Mix (Applied Biosystems in triplicate on the Eco™ real time system (Illumina, San Diego, CA USA). The real-time qRT-PCR results were analyzed and expressed as relative expression of threshold cycle value that was then converted to x-fold changes (2^−∆∆ Ct^) following manufacturer’s instructions and primer sets (listed in Table 1).

For miR-335 expression analysis, the corresponding miR-335 (Assay ID: 000546) and RNU6B (Assay ID: 001093) primers and TaqMan^®^ MicroRNA assay was performed following the manufacturer’s instructions (Applied Biosystems). RNU6B served as an endogenous control for normalization. Real-time qRT-PCR was conducted at 95 °C for 10 min, 40 cycles of 95 °C for 15 s and 65 °C for 1 min. Stem loop sequence of miR-335 and RNU6B are described in Table 2.

### Western blotting

Cells were harvested using RIPA buffer (Thermo Scientific, Waltham, MA, USA) supplemented with a protease inhibitor cocktail (Roche). The protein concentrations were determined by using the Pierce BCA Protein Assay Kit (Thermo Scientific). Western blotting was performed as described earlier^35^ using following antibodies: Anti-p16 (C-20), anti-p21 (C-19), anti-p53 (DO-1), anti-MDM2 (HDM2-232), anti-Cdk4 (C-22), anti-E2F (MH5), anti-GFP (B-2) (Santa Cruz, CA, USA), anti-Phospho-Rb (Ser780), anti-Rb (4H1) (Cell Signaling, Danvers, MA, USA) and anti-CARF (FLA-10)^52^. Anti-β-actin antibody (AC-15) (Abcam, Cambridge, MA, USA) was used as an internal loading control. All the experiments were performed in triplicate at least three times.

### Immunostaining

Cells were cultured on a glass coverslip placed in a 12-well culture dish. For immunostaining, cells wee washed (cold PBS), fixed (pre-chilled methanol/acetone (1:1) mixture for 10 min), permeabilized (0.5% Triton X-100 in PBS for 10 min), blocked (0.2% BSA/PBS for 1 h) and were then incubated with specific antibody (as described above) for overnight at 4 °C. They were incubated with Alexa-594-conjugated goat anti-mouse or anti-rabbit (Molecular Probes, Invitrogen) secondary antibodies after washings (thrice) with 0.2% Triton X-100 in PBS. Counterstaining was performed with Hoechst 33342 (Sigma) for 10 min in dark following by extensive washings with 0.2% Triton X-100 in PBS and observations under a Carl Zeiss microscope (Axiovert 200 M, Tokyo, Japan)^35^. At least 200 cells (from 2–5 random fields in view) were evaluated for quantitation of immunofluorescence signals. Images were quantified by ImageJ software (National Institute of Health, Bethesda, MD).

### *In vivo* tumor formation assay

Balb/c nude mice (4 weeks old, female) were provided by the Institute of Laboratory Animal Science of Peking Union Medical College. Cells (A549 and A549-miR335 transfected cells.) were injected into the nude mice subcutaneously (5 × 10^6^ suspended in 0.2 ml of growth medium). Tumor formation and mice health (body weight) was monitored every alternate day. The experiment was repeated twice, using 4 mice in each group. Volume of the subcutaneous tumors was calculated as V = L × W^2^/2, where L was length and W was the width of the tumor, respectively. Protocols for animal experiments were approved by the Animal Care and Use Committee, Institute of Laboratory Animal Science of Peking Union Medical College (ILAS-PG-2014-018).

### Statistical analysis

All experiments were carried out more than three times, and data were expressed as mean ± standard deviation (SD). Data were represented with respect to the control that were set either at 100 or 1 as shown. Two-tailed Student’s t-test or nonparametric ManneWhitney U-test, whichever was applicable, was used to determine the degree of significance between the control and experimental sample. Statistical significance was defined as significant (^*^p-value ≤ 0.05), very significant (^**^p-value ≤ 0.01) and very very significant (^***^p-value ≤ 0.01).

## Additional Information

**How to cite this article**: Yu, Y. *et al*. Loss-of-function screening to identify miRNAs involved in senescence: Tumor suppressor activity of miRNA-335 and its new target CARF. *Sci. Rep.* **6**, 30185; doi: 10.1038/srep30185 (2016).

## Supplementary Material

Supplementary Information

## Figures and Tables

**Table 1:** qRT-PCR primer set sequences.

Gene (human)	Sequence (5′-3′)
p21^WAF1^ forward	GAGGCCGGGATGAGTTGGGAGGAG
p21^WAF1^ reverse	CAGCCGGCGTTTGGAGTGGTAGAA
CDK4 forward	TCGAAAGCCTCTCTTCTGTG
CDK4 reverse	TACATCTCGAGGCCAGTCAT
p16^INK4a^ (1) forward	CCCAACGCACCGAATAGTTA
p16^INK4a^ (1) reverse	ACCAGCGTGTCCAGGAAG
p16^INK4a^ (2) forward	GAAGGTCCCTCAGACATCCCC
p16^INK4a^ (2) reverse	CCCTGTAGGACCTTCGGTGAC
p16^INK4a^ (3) forward	CCCCTTGCCTGGAAAGATAC
p16^INK4a^ (3) reverse	AGCCCCTCCTCTTTCTTCCT
Rb forward	GGAAGCAACCCTCCTAAACC
Rb reverse	TTTCTGCTTTTGCATTCGTG
p53 forward	TAACAGTTCCTGCATGGGCGGC
p53 reverse	AGGACAGGCACAAACACGCACC
HDM2 forward	TAGTATTTCCCTTTCCTTTGATGA
HDM2 reverse	CACTCTCCCCTGCCTGATAC
CARF forward	TCAAAGTGACAGATGCTCCA
CARF reverse	CGTTGAACTGTTTTCCTGCT
18s forward	CAGGGTTCGATTCCGTAGAG
18s reverse	CCTCCAGTGGATCCTCGTTA

**Table 2:** Stem loop sequence.

miR-335	UGUUUUGAGCGGGGGUCAAGAGCAAUAACGAAAAAUGUUUGUCAUAAACCGUUUUUCAUUAUUGCUCCUGACCUCCUCUCAUUUGCUAUAUUCA
RNU6B	GCAAGGATGACACGCAAATTCGTGAAGCGTTCCATATTTTT
