# Supplementary material for: Loss-of-function screening to identify miRNAs involved in senescence: tumor suppressor activity of miRNA-335 and its new target CARF
Source: Sci Rep. 2016 Jul 26;6:30185. doi: 10.1038/srep30185 (PMC4960484; doi:10.1038/srep30185)
Supplement: Supplementary Information [file srep30185-s1.pdf]

Loss-of-function screening to identify miRNAs involved in senescence: tumor suppressor activity of miRNA-335 and its new target CARF

Yue Yu[1,2,*], Ran Gao[1,3,*], Zeenia Kaul[4], Ling Li[1,2], Yoshio Kato[1], Zhenya Zhang[2], Joanna Groden[4], Sunil C Kaul[1] & Renu Wadhwa[1]

[1]Drug Discovery and Assets Innovation Lab, DBT-AIST International Laboratory for Advanced Biomedicine (DAILAB), Biomedical Research Institute, National Institute of Advanced Industrial Science & Technology (AIST), Tsukuba - 305 8565, Japan. [2]Graduate School of Life & Environmental Sciences, University of Tsukuba, Japan. [3]Institute of Laboratory Animal Science, Chinese Academy of Medical Science (CAMS) & Comparative Medicine Center, Peking Union Medical College (PUMC), China. [4]Department of Molecular Virology, Immunology & Medical Genetics, The Ohio State University, Columbus, Ohio 43210, USA.

Running title: Tumor suppressor function of miRNA-335

[*]These authors contributed equally to this work.

Correspondence and requests for materials should be addressed to S. C. K. (email: s-kaul@aist.go.jp) or R. W. (email: renu-wadhwa@aist.go.jp)

A

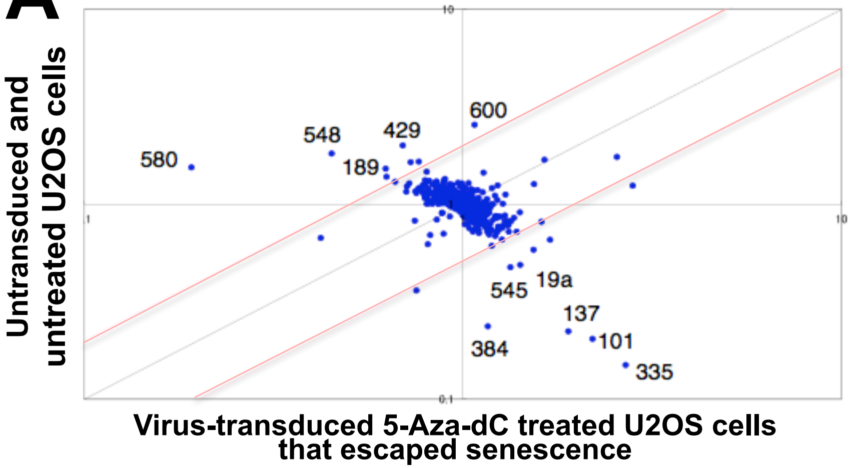

B

miRNA	Fold increase
H-335	17.95
H-101	10.78
H-137	8.52
H-384	4.92
H-19a	2.90
H-545	2.81
H-let-7g	2.62
H-558	2.58
H-18b	2.25
H-144	2.08

C

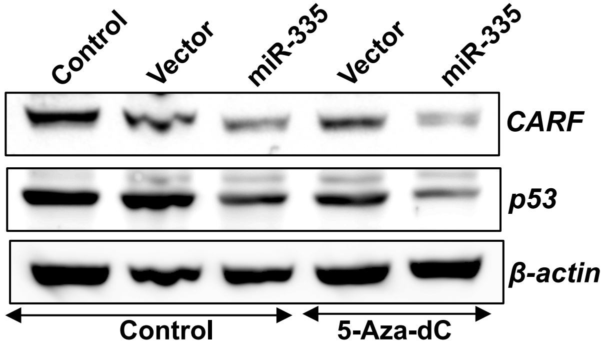

E

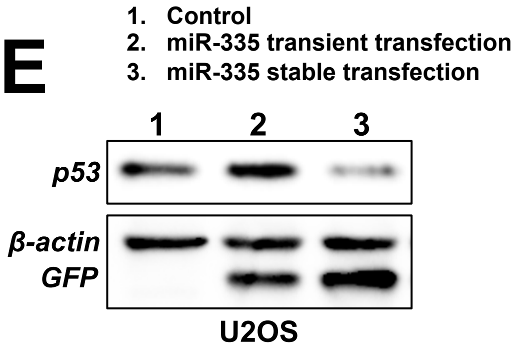

D

	Predicted consequential pairing of target region (top) and miRNA (bottom)	Site type
Position 2347-2354 of CDKN2AIP 3' UTR<br>hsa-miR-335-5p	5' ...AGAGAGCCGUGUUCAGCUCUUGA...<br>||| ||||||| 3' UGUAAAAAGCAAUAA-CGAGAACU	8mer
Position 3110-3116 of CDKN2AIP 3' UTR<br>hsa-miR-335-5p	5' ...AAAACCUAGAAAAAUGCUCUUGG...<br>||||||| 3' UGUAAAAAGCAAUAACGAGAACU	7mer-m8

Supplementary Fig. 1

Scatter plot analysis of miRNA array in which the expression of miRs in 5-Aza-dC treated cells was normalized with untreated cells (**A**). Each dot in the graph represents miR that showed change in expression level on logarithmic scales; x-axis and y-axis show 5-Aza-dC treated and control cells, respectively. Two red diagonal lines indicate the 2-fold cut-off point. miRs showing, at least, 2-fold increase in expression in 5-Aza-dC treated cells that bypassed senescence (**B**) were considered for further analysis, and miR-335 (bottom-rightmost in the graph) that showed the highest (17-fold) upregulation (**B**) is described in this study. Decrease in CARF and p53 expression in miR-335, but not the vector, transfected cells (**C**). miR-335 target site prediction by TargetScanHuman ver. 7.0 (http://www.targetscan.org) [PMID: 26267216]. Predicted binding site for miR-335 in the 3'UTR of CARF (also known as CDKN2AIP) are listed (**D**). p53 expression in cells transfected, either transiently or stably, with miR-335 expression vector. Whereas the transiently transfected cells showed increase in p53, the stably transfected cells showed decrease (**E).**

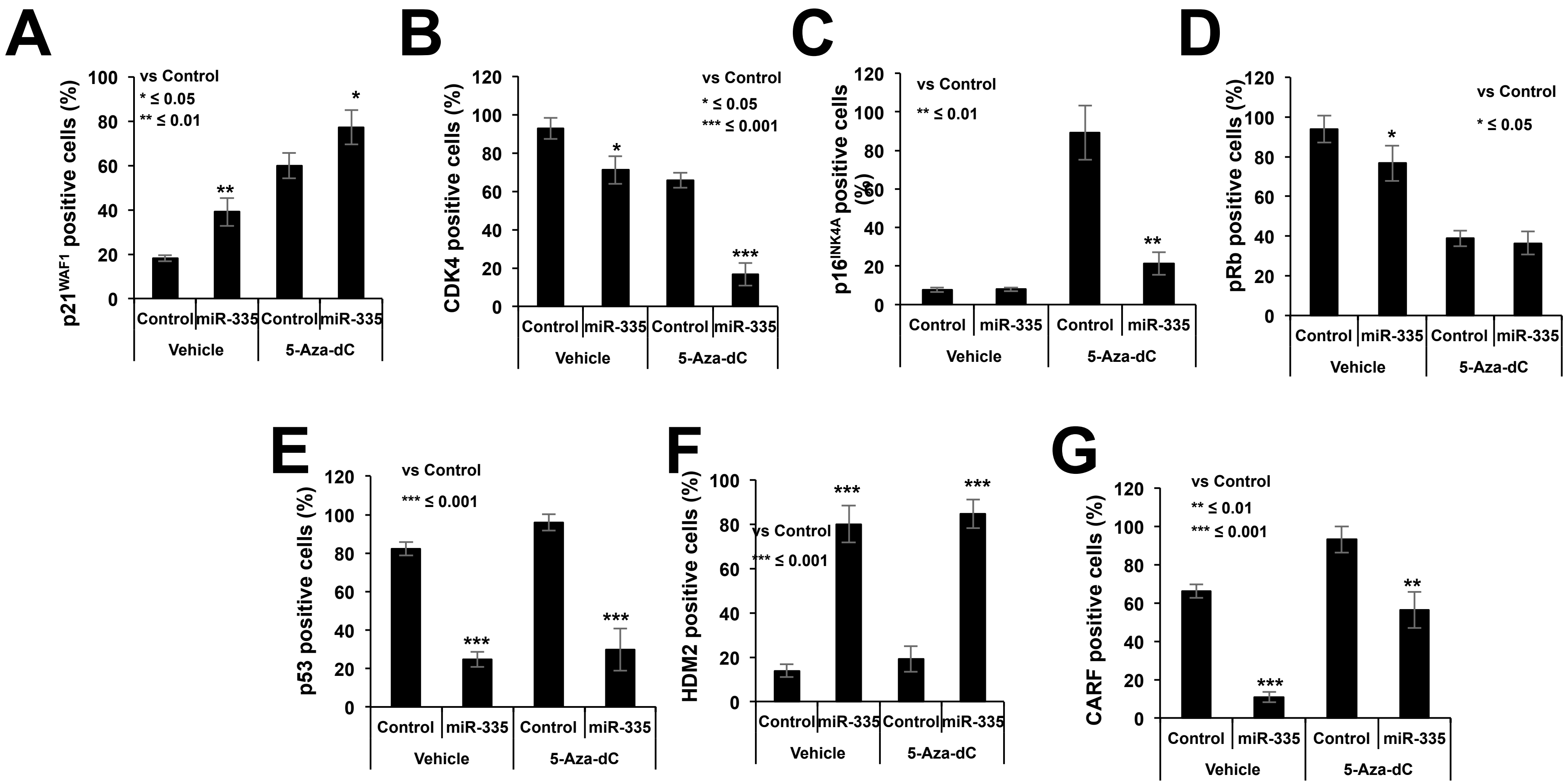
A
vs Control
* ≤ 0.05
** ≤ 0.01
p21WAF1 positive cells (%)
Control
miR-335
Vehicle
5-Aza-dC
B
vs Control
* ≤ 0.05
*** ≤ 0.001
CDK4 positive cells (%)
C
vs Control
** ≤ 0.01
p16INK4A positive cells (%)
D
vs Control
* ≤ 0.05
pRb positive cells (%)
E
vs Control
*** ≤ 0.001
p53 positive cells (%)
F
vs Control
*** ≤ 0.001
HDM2 positive cells (%)
G
vs Control
** ≤ 0.01
*** ≤ 0.001
CARF positive cells (%)

Supplementary Fig. 2

Quantitation of immunofluorescence signals for $p21^{WAF1}$ (A), CDK4 (B), $p16^{INK4A}$ (C), pRb (D), p53 (E), HDM2 (F) and CARF (G) is shown.
